# Leptin deficiency-caused behavioral change – A comparative analysis using EthoVision and DeepLabCut

**DOI:** 10.3389/fnins.2023.1052079

**Published:** 2023-03-24

**Authors:** Daniel Bühler, Nicole Power Guerra, Luisa Müller, Olaf Wolkenhauer, Martin Düffer, Brigitte Vollmar, Angela Kuhla, Markus Wolfien

**Affiliations:** ^1^Rudolf-Zenker-Institute for Experimental Surgery, Rostock University Medical Center, Rostock, Germany; ^2^Institute of Experimental Epileptology and Cognition Research, University Medical Center Bonn, Bonn, Germany; ^3^Department of Systems Biology and Bioinformatics, University of Rostock, Rostock, Germany; ^4^Clinic and Polyclinic for Otorhinolaryngology and Otolaryngology, Faculty of Medicine Carl Gustav Carus, Technische Universität Dresden, Dresden, Germany; ^5^Centre for Transdisciplinary Neurosciences Rostock (CTNR), Rostock University Medical Center, Rostock, Germany; ^6^Department of Psychosomatic Medicine and Psychotherapy, Rostock University Medical Center, Rostock, Germany; ^7^Leibniz-Institute for Food Systems Biology, Technical University of Munich, Freising, Germany; ^8^Institute for Medical Informatics and Biometry, Faculty of Medicine Carl Gustav Carus, Technische Universität Dresden, Dresden, Germany; ^9^Center for Scalable Data Analytics and Artificial Intelligence (ScaDS.AI), Dresden, Germany

**Keywords:** behavioral analysis, obesity, EthoVision, DeepLabCut, deep learning

## Abstract

**Introduction:**

Obese rodents e.g., the leptin-deficient (ob/ob) mouse exhibit remarkable behavioral changes and are therefore ideal models for evaluating mental disorders resulting from obesity. In doing so, female as well as male ob/ob mice at 8, 24, and 40 weeks of age underwent two common behavioral tests, namely the Open Field test and Elevated Plus Maze, to investigate behavioral alteration in a sex- and age dependent manner. The accuracy of these tests is often dependent on the observer that can subjectively influence the data.

**Methods:**

To avoid this bias, mice were tracked with a video system. Video files were further analyzed by the compared use of two software, namely EthoVision (EV) and DeepLabCut (DLC). In DLC a Deep Learning application forms the basis for using artificial intelligence in behavioral research in the future, also with regard to the reduction of animal numbers.

**Results:**

After no sex and partly also no age-related differences were found, comparison revealed that both software lead to almost identical results and are therefore similar in their basic outcomes, especially in the determination of velocity and total distance movement. Moreover, we observed additional benefits of DLC compared to EV as it enabled the interpretation of more complex behavior, such as rearing and leaning, in an automated manner.

**Discussion:**

Based on the comparable results from both software, our study can serve as a starting point for investigating behavioral alterations in preclinical studies of obesity by using DLC to optimize and probably to predict behavioral observations in the future.

## Introduction

Abnormal and excessive accumulation of fat tissue associated with severe overweight and obesity is one of the most challenging diseases of the 21st century (World Health Organization [WHO], 2021). Obesity is a public health concern affecting both genders at all ages around the world and is an enormous burden on the global health system due to an increasingly high prevalence. Obesity-associated comorbidities are mostly diabetes type 2 (Drucker, 2021), cardiovascular diseases (Powell-Wiley et al., 2021), and vascular diseases that can affect function and quality of life and thus can cause mental disease (Sarma et al., 2021). The most common current psychiatric disorders in obese patients are depression and anxiety (Moradi et al., 2021). It is still largely unclear to what extent obesity affects mental health. Obese rodents remarkably exhibit behavioral alterations such as anxiety and are therefore effective models in behavioral research (Guma et al., 2022) to further address research questions on mental health. A transgene model for obesity research is the leptin deficient mouse (ob/ob) (Zhao et al., 2020). Animal behavior can be assessed by using different types of behavioral tests, such as the Open Field (OF) test and the Elevated Plus Maze (EPM). Both tests are among the most commonly used methods in behavioral laboratories to investigate changes in anxious-related behavior in rodents (Rodgers and Shepherd, 1993). For instance, reduced exploration (rearing/head dipping) indicates anxiety, and reduced locomotion has been linked to adaptive stress-related behaviors (Walf and Frye, 2007). To record such behavior, previously, this would be done live by a trained observer, scoring (i.e., classifying) the behavior of the animal live while the test was being conducted. Nowadays, the standard approach is to record the complete test session with high-speed cameras, which are positioned above the testing arenas as to capture all behavior of the animal. Subsequently, the behavior is being scored *post hoc* by analyzing the videos (CVPR 2019 Open Access Repository, 2019). Traditional approaches to analyze video data and score the animals behavior usually involved several researchers watching videos of behavioral test sessions and noting the times and locations of specific events of interest. Although previously seen as fold-standard, these investigations have been very time-consuming, needed professional knowledge of ethologists, and were prone to be influenced by potential observer bias (Datta et al., 2019). In order to produce behavioral data that is comparable across laboratories, it is important to develop standardized methods that are robust, reproducible and minimize the influence of biases. Therefore, semi-automated video analysis methods have been developed decades ago. However, only the latest developments in artificial intelligence (AI) and ever faster computing power enable it to evaluate videos automatically and reduce the bias by human observers even further (Valletta et al., 2017).

Advances in automatic video analysis make it possible to analyze animal behavior independently of an observer (Sturman et al., 2020). Commercial products, such as EthoVision (EV, Noldus, Wageningen, Netherlands), are available for semi-automated tracking of behavior in videos. However, these systems have some limitation, for example they depend on the ability to detect the subject based on differences of background color and cannot account for dynamic aspects of behavior. EV enables animal tracking in a wide range of animal testing batteries and is independent of visible or artificially applied markers, as it calculates the central point of the torso on the basis of the contour of the moving mouse (Sturman et al., 2020). To analyze the movement, this point is recalculated frame by frame. EV is widely used in behavioral analysis because it offers an easy-to-use interface and can be installed on common computers in almost any laboratory. Accordingly, Stringer et al. (2019) investigated spontaneous coding of visual signals and studied motion-related information in mouse visual cortex using such an approach. Moreover, Musall et al. (2019) analyzed cortical imaging data using multiple discrete measures of performance (e.g., stimulus type and reaction time) and video-based measures of movement from mice performing a decision-making task. While these approaches have led to novel insights into neural determinants of behavior, they are limited to categorize movements, measure transitions between different types of movements, or quantify the dynamics of movement sequences (Musall et al., 2019). Recent developments in the field of computer vision and machine learning (ML) offer a solution to measure these behavioral dynamics. As a result, first descriptions of unsupervised behavioral analyses revealed astonishing temporal and structural complexity of rodent behavior. However, these advanced analyses are not applicable for many behavioral research laboratories, as they usually lack necessary hardware and needed know-how. To overcome this issue, a growing number of research groups develop methods that enable an analysis of motion and associated-behavior more efficiently, and above all, automatically (Knutsen et al., 2005; Voigts et al., 2008; Perkon et al., 2011; Clack et al., 2012; Ohayon et al., 2013; Giovannucci et al., 2018; Dominiak et al., 2019; Vanzella et al., 2019; Betting et al., 2020; Petersen et al., 2020). Recently, Deep Learning (DL) applications have been used in behavior imaging data analysis. Most commonly used for this purpose are so-called convolutional neural networks (CNN), a class of deep neural networks. They consist of nodes (“neurons”), including with learnable weights and biases that have been trained from the input data (“edges”). In particular, the development of DeepLabCut (DLC), which is a markerless pose-estimation toolkit based on DL (Mathis et al., 2018), facilitates the generation of networks with very small training sets. Using DLC, it is now possible to score behavior completely independent of an observer and allows the tracking of animal movements of nearly any part of the body. In turn, it offers the possibility of uncovering new behavioral patterns with the help of unsupervised learning approaches. This could lead to the identification of causal mechanisms of various diseases that are reflected in behavioral changes.

Herein, this study was conducted to compare two common pose estimation methods, namely EV and DLC, to characterize obese-related behavioral changes in ob/ob mice. In addition, this preclinical research should help to clarify the extent to which behavioral changes can be covered just as well or even better with DLC in order to predict behavior with AI in the future.

## Materials and methods

### Animals

**FIGURE 1:**
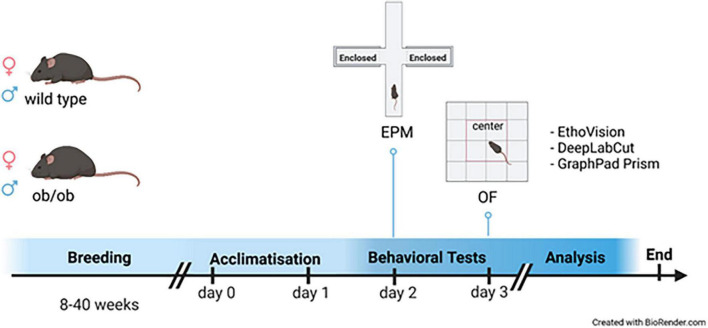
Experimental design. Graphical illustration of the experimental procedure; Male (♂ blue) and female (♀ red) leptin deficient (ob/ob) and wild type (wt) at the age of 8, 24, and 40 weeks were firstly habituated to the testing room on day 1 for 24 h; behavioral tests were conducted on day 2 [Elevated Plus Maze (EPM)] and 3 [Open Field (OF)]. After behavioral tests were conducted the animals were killed to obtain physiological data. All behavioral data were analyzed with both EthoVision (EV) and DeepLabCut (DLC), and statistically evaluated with GraphPad Prism and Excel. The graphic was created with BioRender.com.

For the study, 45 (15 male and 30 female) leptin deficient mice (ob/ob) and 46 (13 male and 33 female) wild type (wt, C57BL6) were used. The animals were littermates at the age of 8, 24, and 40 weeks. They were housed in standard cages with up to 5 animals per cage, in a temperature-controlled room (21 ± 3°C) with a 12/12 h day-night cycle (lights from 06:00 a.m. to 06:00 p.m.) containing a twilight period of 30 min. The exact number of the corresponding age groups is given in Table 1. Both wt and ob/ob mice were generated from our own breeding and were fed the same standard diet (ssniff R/M-H, ssniff^®^ Spezialdiäten GmbH, Soest, Germany). All animal experimental work was carried out with permission of the local Animal Research Committee [Landesamt für Landwirtschaft, Lebensmittelsicherheit, und Fischerei (LALLF)] of the state Mecklenburg-Western Pomerania (LALLF M-V/TSD/7221.3-2-001/18, approved on 1 March 2018) and all animals received human care according to the EU Directive 2010/63/EU. The experimental design is illustrated in Figure 1.

**TABLE 1:** Amount of animals.

Age of animals	Female (*n*)		Male (*n*)	
	**wt**	**ob/ob**	**wt**	**ob/ob**
8 weeks	10	10	3	5
24 weeks	10	10	5	5
40 weeks	13	10	5	5
	**33**	**30**	**13**	**15**

The groups were arranged based on age, sex, and genotype. wt, wild type; ob/ob, leptin deficient mice. The bold numbers indicate the respective sums of animals for the individual column.

### Behavioral procedures

Behavioral analyses were performed on wt and ob/ob mice with no prior handling other than routine husbandry. Experiments were conducted between 07:00 a.m. and 03:00 p.m. under normal lighting conditions. The same operator (NPG for female mice; DB for male mice) assessed the mice aged 8, 24, and 40 weeks. All age groups and both sexes were used for the behavioral tests. Mice were brought into the testing room in their home cages 120 min prior to testing to allow adaptation. Each animal received a single trial, 5 min for both OF and EPM. Between both tests, breaks with a length of at least 1 day in the testing room were carried out. The behavior was recorded using a video camera system (Camera CCA1300-60 mg, Basler, and lens 15E, Computar, Japan) located 100 cm above the box and maze, and the EthoVision (EV) XT 11.5 software (Nodulus Information Technology). For more details please see Power Guerra et al. (2021).

### Open field (OF)

Curiosity, exploratory, and locomotor activity of wt and ob/ob mice were assessed in a well illuminated, 50 cm × 50 cm squared plastic box, which was virtually divided into 16 zones by a 4 × 4 grid formation. Each zone was 12.5 cm × 12.5 cm. The walls were 40 cm high. For each trial the mice were placed into the center of the arena and were allowed to explore the field for 5 min. Next, each mouse was removed and placed back into the home cage. The total distance (cm), velocity (cm/s), visits in center (n) and periphery (n) were analyzed.

### Elevated plus maze (EPM)

The EPM is a behavioral test to investigate unconditioned anxiety-related behavior that involves a conflict between the desire to explore a novel environment and anxiogenic elements, such as elevation and a brightly illuminated arena. The EPM was made from gray poly vinyl chloride, according to the description of Komada et al. (2008). The 60 cm high elevated platform consists of two open arms measuring 37 cm × 6 cm (length × width) and perpendicular to the open arms were two closed arms of the same dimension, but with additional walls of 12.4 cm high. The cross at the center of the four arms consisted of a 6 cm × 6 cm square. The open arms contained a slight ledge (4 mm) to prevent mice from falling off the arms.

Each mouse was placed into the center square facing the open arm most distant to the experimenter and was allowed to freely move and explore the EPM for 5 min. Next, each mouse was removed and placed back into the home cage. Total distance (cm), velocity (cm/s), visits in open arm (n) and closed arms (n) were measured.

### Video analysis with the EthoVision XT 11.5 software

In addition to analysis of videos, EV (EthoVision XT 11.5) was used to record all OF and EPM videos. The in-build “Automatic animal detection settings” of EV were used for all video analysis. Slight tuning of these settings was performed using the fine-tuning slider in the automated animal detection settings to ensure the animals could be tracked throughout the entire arena. We ensured there was a smooth tracking curve and that the center point of the animal remained stable before analysis took place. The automated tracker option of EV classified arm entries based on the nominal center of a mouse crossing into an arm. The rearing counts were collected manually by video analysis.

### Video analysis with DeepLabCut

In addition to EV analysis, also DLC was used to evaluate the behavior of the animals in the OF and EPM. For this purpose, the same videos from the EV analysis were used. Data generated by DLC was processed using customized R Scripts that are available online.^1^ The data acquisition workflow can be seen in the Supplementary Figure 1 [for more detailed information see Sturman et al. (2020)]. In brief, DLC was used to track 13 body points of interest (Supplementary Figure 2), and the corner points of the OF/EPM arena, since these are also recommended default parameters for subsequent analysis tools, e.g., SimBa (Nilson et al., 2020). To train the networks for both tests, 20 frames from multiple, randomly selected videos were selected and run for ∼500.000 iterations. Subsequently, the coordinates obtained were analyzed with an adapted R GitHub script,^2^ which was initially provided by ETH Zürich. Values of tracked points with low accuracy (<0.95) were removed. The velocity and distance of each point was determined by estimating the animal’s position over 5 min.

### Server specifications

The DLC computations have been utilized by a CentOS Linux compute node equipped with 8 × Nvidia Quadro RTX 2080 Ti, 2 × Intel Xeon Gold 6142 (16 Cores, 32 Threads), and 768 GB RAM.

### Weight control and euthanasia

Body weight was measured (Kern PCB, Lübeck, Germany) prior to euthanasia. Under anesthesia (5 vol. % isoflurane; Baxter, Unterschleißheim, Germany) the mice were euthanized, laparotomized, and visceral and subcutaneous flanked fat deposits were harvested and weighed (Power Guerra et al., 2021).

### Statistics

Statistical analysis was performed using GraphPad Prism 8.0.1 (GraphPad Software Inc., San Diego, CA, USA) or Excel. The ROUT method based on false discovery rate (*Q* = 0.01) was used to identify and re-move outliers if possible and necessary. Data were also excluded when mice did not perform behavioral tests. Data were tested for normal distribution and all comparisons between normally distributed datasets containing two independent groups were performed using unpaired Student’s *t*-test, whereas all comparisons with more than two groups were performed using two- or three-way ANOVAs in order to identify group effects. Significant main effects were then followed up with *post-hoc* tests. Further information is given in respective Figure legends. The results are presented as box plots indicating the median, the 25th and 75th percentile in the form of a box with the confidence interval of 95%. To represent correlation between DLC and EV software (Figure 5 and Supplementary Figure 8). Spearman Correlation was performed by using confidence interval of 90% and measuring linear dependence of two parameters. Additionally, a Bland-Altman Plot analysis was performed to evaluate the agreement between the used methods (EV and DLC).

## Results

### Leptin deficiency affects massive body weight gain

**FIGURE 2:**
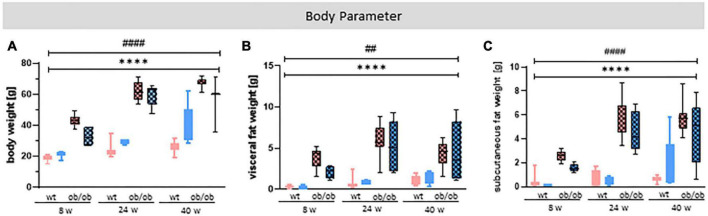
Statistical investigation of different body parameters to characterize obesity. **(A)** Body weight (g), **(B)** visceral, and **(C)** subcutaneous fat weight (g) of female (red) and male (blue) mice at age of 8, 24, and 40 weeks were analyzed at the end of the experiment. The group size was as follows: *n*(wt) = 45 **(A)**, 41 **(B)**, 33 **(C)**, *n*(ob/ob) = 42 **(A,C)**, 45 **(B)**. Significances of differences between the groups were tested by three-way ANOVA. The analysis revealed a genotype and age, but no sex effect for all three examined parameters. Statistical significance was set at ****p < 0.0001 for genotype effect and ^##^*p* < 0.01 or ^####^*p* < 0.0001 for age effect. ob/ob, leptin deficient mice; wt, wild type; w, weeks (these abbreviations will be consistently used in all following plots, unless stated otherwise).

Body weight (Figure 2A), visceral fat (Figure 2B), and subcutaneous fat (Figure 2C) were significantly increased in ob/ob mice compared to the control wt littermates by data showing a strong effect of genotype [A: *p* < 0.0001, *F*_(1,75)_ = 508.6; B: *p* < 0.0001, *F*_(1,74)_ = 104.2; C: *p* < 0.0001, *F*_(1,63)_ = 148.7]. In addition, the data also show a age effect [A: *p* < 0.0001, *F*_(2,75)_ = 69.92; B: *p* = 0.0041, *F*_(2,74)_ = 5.940; C: *p* < 0.0001, *F*_(2,63)_ = 14.06], but no sex effect (Figure 2).

### Sex does not and age only partly impact behavioral alteration

**FIGURE 3:**
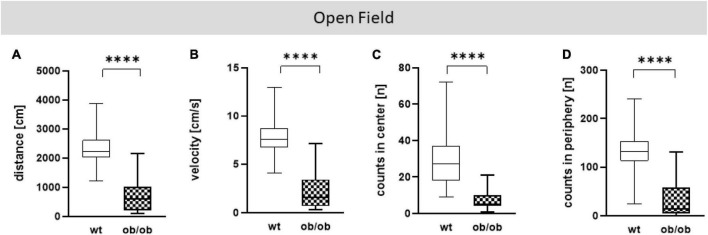
Open Field (OF) test analysis. **(A)** Moved distance (cm), **(B)** mean velocity (cm/s), **(C)** number of visits (counts) in center (n), **(D)** number of visits (counts) in periphery (n). Analyses were done with the EthoVision software. The group size was as follows: *n*(wt) = 36 **(A–D)**, *n*(ob/ob) = 40 **(A,B,D)** and 39 **(C)**. Significance of differences between the groups were tested by unpaired Student’s *t*-test. Statistical significance was set at ****p < 0.0001.

**FIGURE 4:**
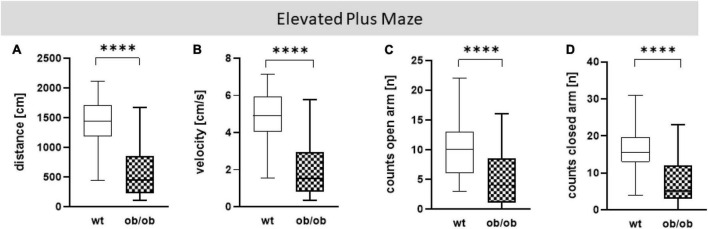
Elevated Plus Maze (EPM) analysis. **(A)** Moved distance (cm), **(B)** mean velocity (cm/s), **(C)** number of visits (counts) on open arm (n), **(D)** number of visits (counts) in closed arm (n). Analyses were done with the EthoVision software. The group size was as follows: *n*(wt) = 45 **(A–C)** and 44 **(D)**, *n*(ob/ob) = 45. Significance of differences between the groups were tested by unpaired Student’s *t*-test. Statistical significance was set at ****p < 0.0001.

Firstly, we examined the association of sex and age on behavioral alteration, analyzed in the OF (total distance movement, velocity, and counts in center and periphery) and EPM (total distance movement, velocity, and counts in open and closed arms). Statistical analysis including all correlating parameters of female and male ob/ob and wt mice at an age of 8, 24, and 40 weeks revealed that mainly the genotype was responsible for behavioral alteration in ob/ob mice, when compared to their wt littermates. Although statistical analysis revealed still partly an age effect, the obese genotype already showed significant changes at 8 weeks of age in comparison to wt mice, therefore age seems to play a minor role in obesity-related behavior alteration in the ob/ob mice. Concerning general locomotor activity, both female and male ob/ob as well as wt mice at age of 8, 24, and 40 weeks displayed no difference, neither in traveled distance nor in mean velocity in the OF, as can be seen in Supplementary Figure 3 (three-way ANOVA analysis revealed significant genotype and age, but no sex effect). Likewise, both sexes at all age groups spent approximately the same time in the center or peripheral zone in the OF (Supplementary Figure 4, three-way ANOVA analysis revealed significant genotype and partly age, but no sex effect). Correspondingly, both sexes at all age groups displayed the same behavior analyzed in the EPM (Supplementary Figures 5, 6, three-way ANOVA analysis revealed significant genotype and partly age and sex effect). Hence, for further *post hoc* analysis, both sex and age datasets were merged. In doing so, collected data in the OF test of total distance movement and velocity were significantly decreased for ob/ob mice when compared to wt mice (Figures 3A, B, *p* < 0.0001). Similarly, times spent in the center, as well as in peripheral zone, were significantly reduced for ob/ob vs. wt mice (Figures 3C, D, *p* < 0.0001). Correspondingly, in EPM significant differences in traveled distance and velocity (Figures 4A, B, *p* < 0.0001) were displayed between both mice strains. In addition, ob/ob mice visited significantly less open arms and closed arms in comparison to wt mice (Figures 4C, D, *p* < 0.0001).

**FIGURE 5:**
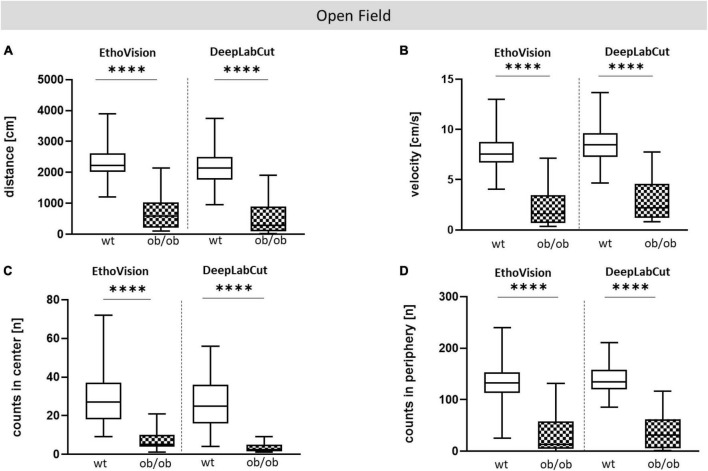
Comparative analysis with EV and DLC for OF test. OF analysis of wt and ob/ob mice. **(A)** Moved distance (cm), **(B)** mean velocity (cm/s), **(C)** number of visits (counts) in center (n), **(D)** number of visits (counts) in periphery (n). Significance of differences between the groups were tested by unpaired Student’s *t*-test. Group size was as follows: *n*(wt) = 36(EV) or 35(DLC), *n*(ob/ob) = 40 [EV **(A,B,D)**] and 39 **(C)** or 38 [DLC **(A,B,D)**] and 34 **(C)**. Statistical significance was set at ****p < 0.0001. EV, EthoVision; DLC, DeepLabCut.

### DeepLabCut achieves comparable results to EthoVision

#### Precise animal tracking

In order to establish an alternative to the commercial tracking software EV, EV was compared with the more flexible and open source available software DLC. The developers of DLC have already demonstrated the robustness and precision of the tracking (Mathis et al., 2018). For this reason, we used DLC tracking in arenas compatible with commercial systems (such as EV) that we regularly use in our laboratory. To test for equivalent usability, data from the most commonly used tests in behavioral research, the OF and EPM, were used, and the results of the two different tools –EV and DLC- were cross-referenced.

#### Open field and elevated plus maze

To compare DLC-tracking performance with EV, data from OF and EPM behavioral tests that were collected with EV were included, and reused to create video files for training DLC models. Locomotor parameters of the OF and EPM analysis, such as total distance, mean velocity, and number of visits (counts) in predefined zones were comparable between DLC and EV (Figures 5A–D for OF; Supplementary Figure 8 for EPM). Both tools showed significant differences between ob/ob and wt mice (distance and velocity, *p* < 0.0001; counts in center and periphery, *p* < 0.0001) for OF and (distance and velocity, *p* < 0.0001; counts in open arm and closed arm, *p* < 0.0001) for EPM. Spearman correlation analysis of OF and EPM data-set further revealed that both software tools showed a high comparability, which was almost significant (Supplementary Figures 7, 9). Moreover, the descriptive analysis of Bland-Altman showed that both methods revealed a good agreement (Supplementary Figures 7, 9).

### AI-supported video analysis with DLC enables advanced parameter assessment

**FIGURE 6:**
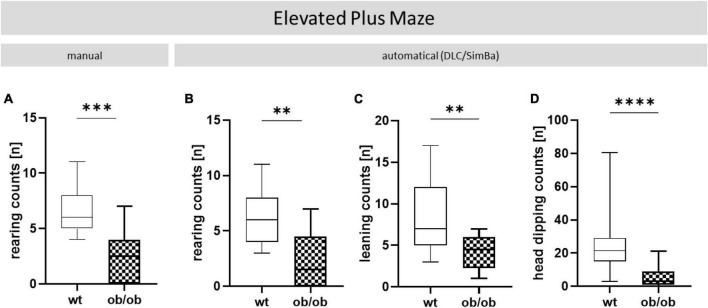
Additional parameters collected in the EPM. Wt and ob/ob mice were comparatively analyzed. **(A)** Rearing counts (n) manually scored by an observer *via* video analysis and **(B)** obtained automatically with DeepLabCut (DLC). Furthermore, **(C)** leaning (n), and **(D)** head dipping counts (n) were observed with DLC. Group size was as follows: *n*(wt) = 11 **(A–C)**, 46 **(D)** and, *n*(ob/ob) = 12 **(A)**, 10 **(B)**, 8 **(C)**, and 42 **(D)**. Significance of differences between the groups were tested by unpaired Student’s *t*-test. Statistical significance was set at ^**^p < 0.01, ^***^p < 0.001, and ****p < 0.0001.

Through the AI-supported video analysis by DLC, different behaviors could also be scored. These included rearing, leaning, and head dipping, which were observed in both behavioral tests. A selection of EPM and OF recordings are accessible at the following link: OF and EPM (see text footnote 2). The above-mentioned parameters had to be recorded manually by direct observation, since the automated evaluation of the videos in EV software did not always detect rearing accurately. However, rearing and further leaning and head dipping could additional obtained automatically by DLC. In doing so, ob/ob vs. wt mice exhibited significantly less rearing-behavior (Figure 6A, *p* < 0.001 and Figure 6B, *p* < 0.01) whereby the manual and the automatical analyses showed equal results. Using pose estimation with DLC, we found in addition that ob/ob mice showed significantly less leaning (Figure 6C, *p* < 0.01) and dipping (Figure 6D, *p* < 0.0001) behavior when compared to wt mice.

**FIGURE 7:**
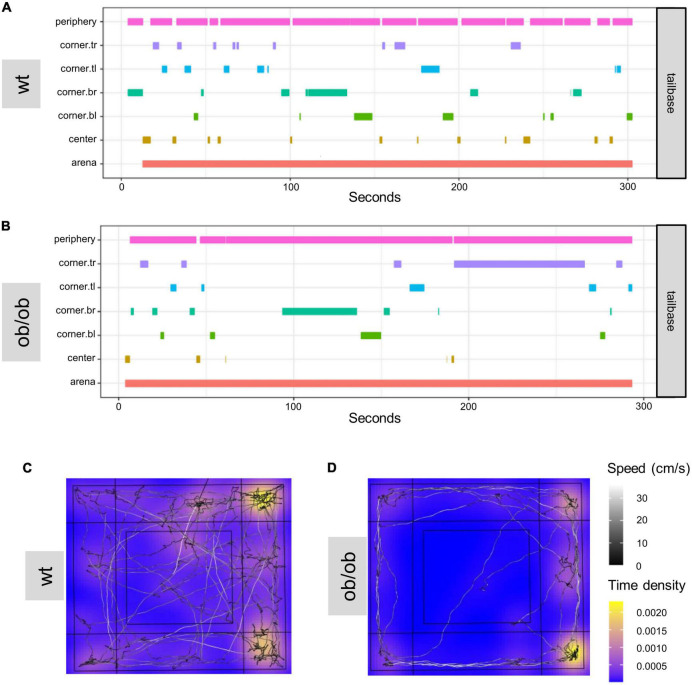
DeepLabCut (DLC) tracking results analyzed with customized R-script based on DLC-Analyzer. Example of a zone-visit-plot of a wild type [**(A)** wt] and an ob/ob **(B)** mouse in OF. The individual zones show how much time the body part of interest (Supplementary Figure 2 for labeling of animals in DLC) was tracked in the predefined zones. Heat-maps of the OF test of wild type [wt **(C)**], and transgene leptin deficient [ob/ob **(D)**] mice. tl, top left; tr, top right; bl, bottom left, br, bottom right.

Furthermore, DLC was used to create zone-visit-plots for individual animals. The plots show how much time one example wt (A) and ob/ob (B) mouse spend in areas of interest during the OF test (Figures 7A, B). In addition, Figure 7 shows representative heat maps of a wt (C) and an ob/ob (D) mouse. The wt mouse showed a normal movement pattern, while the ob/ob mouse hardly moved in the periphery after being placed more often in the corners, which is highlighted yellow.

## Discussion

In the present study, we provided the cognizance that obesity-induced leptin deficiency was already present in early age and was the main cause in mediating anxiety-related behavior whereby the behavioral analyses with EV and DLC show comparable results.

### Behavioral alterations in ob/ob mice are independent of sex and age

Adiposity–and the far worse stage obesity (defined as a BMI of over 30)–is not only a health risk itself, but is often associated with other concomitant and secondary diseases, including mood disorders and emotional diseases (Hamer and Stamatakis, 2012; McCrea et al., 2012). Aguilar-Valles et al. (2015) showed a possible correlation of obesity and neurodegeneration. Furthermore, it is suggested that obesity is associated with cognitive impairment and behavioral alterations (Buie et al., 2019). Therefore, behavioral experiments with animals are important approaches to obtain conclusions about the effect of obesity on emotional states like anxious-related behavior or depression (McCrea et al., 2012).

The transgenic mouse model of leptin deficiency induced obesity (ob/ob mice) leads to a continuous sensation of hunger (Suliman et al., 2019), which causes the animals to continually search for food. Due to the increased food intake, excessive fat accumulation occurs at a young age (Hayakawa et al., 2018). In order to assess leptin deficiency-induced obesity, weight data of ob/ob and wt animals were analyzed first. As expected, ob/ob mice showed significantly increased body weights compared to wt mice. The values for both ob/ob mice and wt mice were within the age-, sex-, and genotype-specific, normal range (for more detailed information see Jackson Laboratory). In the context of total body weight gain, a considerably higher proportion of subcutaneous fat was observed in the ob/ob mice, in addition to the increased visceral fat weight at dissection. Beside genetic-induced obesity, it is common to induce obesity also through different diets (Lutz and Woods, 2012). These include the high fat diet (HFD), western diet (WD), and cafeteria diet (CFD), which are also discussed to be responsible for the sharp increase in the obesity pandemic in humans (Sampey et al., 2011). These diets have a common feature: they lead to excess energy intake due to an unbalanced composition of fat and sugar, which in combination with reduced exercise facilitates obesity (Bracke et al., 2019a,b; Picone et al., 2020). However, applying these diets in wild type rodent models is time-consuming and takes several weeks and even months to take effect of an obese phenotype. To overcome this issue, we used the ob/ob mice knowing to reveal a significant weight gain already at young age as verifying in the present study. Therefore, this mouse model is ideal for studying obesity at a younger age. Due to this, it became apparent in the study that the time points of 24 and 40 weeks were not strictly necessary, since a pronounced adiposity was already present in 8-week-old mice, which ultimately reduces the stress of adiposity with increasing age.

Our study contributes to the overall assumption that obesity, in addition to the common comorbidities, causes behavioral alterations (Finger et al., 2011; Reverte et al., 2012; Alonso-Caraballo et al., 2019). Several related studies also show that in both the genetically induced obesity models and in diet-induced obesity models, particularly locomotion is altered (Collins et al., 2015; Zieba et al., 2019). This was demonstrated by López-Taboada et al. (2020) showing obesity-induced by a WD is associated with reduced locomotion. The same conclusion was reached by Yokoyama et al. (2020), who studied the effect of a HFD, and by Teixeira et al. (2020), who linked the CFD diet to behavioral alteration. A similar situation can be observed in humans (Bracke et al., 2019a). However, it is not yet clear whether an increased weight *per se* leads to a reduction in locomotion, or whether there are other contributing factors on a neuronal or emotional level (Shafizadeh et al., 2021). Nevertheless, the present findings confirm that obesity is indeed associated with a behavioral alteration. Interestingly, our study showed that sex and partly age had no impact (even though statistical analysis revealed an age effect) on the behavior seen in ob/ob mice. Additionally, the finding that sex had no influence on the behavioral change means that both male and female mice can be included in the present and future studies. This reduces the number of animals needed to generate statistically sound results, as both sexes can be used equally. Moreover, it allows for more efficient use of breeding times, as less time is needed to generate a sufficient number of genetically identical mice (promoting the 3R-Principle of Reduction, Russell and Burch, 1959). Furthermore, independence of age means that experiments do not have to be carried out for an excessively long time, since at least in the ob/ob model a significant change in behavior is already visible at a very young age [promoting the 3R-Principle of Refinement, Russell and Burch (1959)].

### Behavior analyses with EV and DLC have comparable results

The challenge that manual video analysis are prone to bias by the observer led to the development of easy-to-use, widely accessible semi-automated computer applications that can be deployed in almost any ethology laboratory, facilitating robustness and reproducibility of research findings. Previous studies have already investigated the benefits of AI-based evaluation methods compared to standard methods (Sturman et al., 2020). However, there has not yet been a study that used AI methods in a mouse model of leptin deficiency. Our work builds upon findings of other research groups to uncover the pathological accumulation of adipose tissue, the resulting and associated behavioral change in the mouse model using AI and could open up new approaches to study pathological conditions induced by malnutrition.

The collected data of the behavioral tests OF and EPM were evaluated both with conventional software, namely EV, which is used as a standard in many behavioral laboratories, and with a fairly new AI-based software, namely DLC (Mathis et al., 2020). To evaluate the new, open-source software DLC, the same animal recordings that were analyzed with EV were compared with the findings of DLC. Both methods yielded the same results, i.e., leptin deficiency but not sex or age caused behavioral alterations. From this perspective, DLC might contribute to fewer animals being used in the future, as these methods can predict behavior based on more complex parameters [promoting the 3R-Principle of Reduction, Russell and Burch (1959)]. These side observations show in addition to the fact that the DLC model is very robust with respect to different age groups, sex, and even mouse strains that DLC assures the implementation of the 3R principle (Russell and Burch, 1959). Alongside the advantages of modern methods already mentioned, which allow behavioral analysis to be replicable and almost with human accuracy, the open source software DLC has further advantages over EV (Mathis et al., 2018; Sturman et al., 2020). For instance, DLC is based on the open-source programming language Python, which makes the code more accessible, easier to understand and to adapt it to individual needs. This enables a collection and individualized analysis of more parameters in comparison to EV (where the code is not as accessible). In addition to the common parameters, such as speed, distance, rearing, and leaning, the DLC was also able to record head dipping (Sturman et al., 2020). Decreasing head dipping frequency is associated with increased anxiety, which makes it another important parameter for assessing the anxiety of animals. Previously, this parameter had to be laboriously recorded by hand. In summary, automatic video evaluation with DLC has made this data processing step much faster, however, this kind of behavioral assessment also requires necessary storage capacities and computing power that are not available to every ethological laboratory.

### Future perspective of DLC

One of the greatest advantages of DLC is the broad and fast-growing DLC-community from all possible areas of science that make questions, data, and results publicly accessible (Mathis et al., 2018; Lauer et al., 2021). This facilitates data sharing, as well as the potential reuse of data other laboratories to train and improve ones own networks. DLC also allows multi-animal analyses to be performed in order to study the social behavior of animals (Lauer et al., 2021). This was hardly possible before, or would have required cost intense software updates for exciting commercial hardware. Further side-applications are emerging from collaborations, which use the DLC coordinates data for further analyses. For example, SimBA, a computer framework that enables users to use these pose-estimation approaches in combination with behavioral annotation and generation of supervised machine-learning behavioral predictive classifiers (Nilson et al., 2020). Accordingly, the pipeline was developed for the analysis of complex social behaviors, but also includes the flexibility for users to generate predictive classifiers across other behavioral modalities with minimal effort and no specialized computational background. Besides this huge advantage, it is already possible to explore new animal behavior patterns using unsupervised trained networks. One of the most widely used software programs for this purpose is B-SOiD (Hsu and Yttri, 2021). B-SOiD is an open-source tool to discover and extract behaviors and sub-actions from pose estimation data (Hsu and Yttri, 2021).

## Summary and conclusion

In this investigation, we compared the accuracy of two pose estimation applications, namely EV and DLC in testing obesity-induced behavioral alterations in the ob/ob mice. The compared methods (both are based on the same algorithms) yielded almost identical results, whereby the more current AI-based application DLC allows more flexibility and enables the investigation of additional parameters (e.g., head dipping and leaning). These findings provide new possibilities to analyze rodent behavior in a more consistent and repeatable way, which can be applied in almost every behavioral laboratory with no need of extensive professional computational knowledge. According to these findings, it is conceivable that data from this study will serve as a basis in further analyses to gain more insights into complex behavioral changes and to predict obesity-related behavioral alterations. In addition, the observation the independence of sex and age could also reduce the number of experimental animals needed to a certain degree, and possibly also replace some of them entirely, promoting the 3R-Principle. Taken together, this novel AI approach and the fact that this mouse model of leptin deficiency has been shown to be present obesity-associated behavioral change already in young age might significantly reduce the number of animals for preclinical research in the future.

## Data availability statement

The datasets presented in this study can be found in online repositories. The names of the repository/repositories and accession number(s) can be found below: Full data table and all coding sections can be accessed under https://github.com/DaBue93/Deep-Behaviour-Master-Thesis.

## Ethics statement

This animal study was reviewed and approved by the Animal Research Committee [Landesamt für Landwirtschaft, Lebensmittelsicherheit und Fischerei (LALLF)] of the state Mecklenburg-Western Pomerania.

## Author contributions

AK: conceptualization, funding acquisition, and project administration. NPG: data curation, investigation, and validation. AK and LM: formal analysis. NPG, AK, LM, DB, and MD: methodology. OW and BV: resources. AK and MW: supervision. DB: visualization. DB, AK, and LM: roles/writing—original draft. DB, AK, MW, and LM: writing—review and editing. All authors contributed to the article and approved the submitted version.

## Footnotes

1
https://github.com/ETHZ-INS/DLCAnalyzer

2
https://github.com/DaBue93/Deep-Behaviour-Master-Thesis
