# Supplementary material for: Leptin deficiency-caused behavioral change – A comparative analysis using EthoVision and DeepLabCut
Source: Front Neurosci. 2023 Mar 24;17:1052079. doi: 10.3389/fnins.2023.1052079 (PMC10079875; doi:10.3389/fnins.2023.1052079)
Supplement: Supplementary file 1 [file Data_Sheet_1.pdf]

frontiers

Supplementary Material

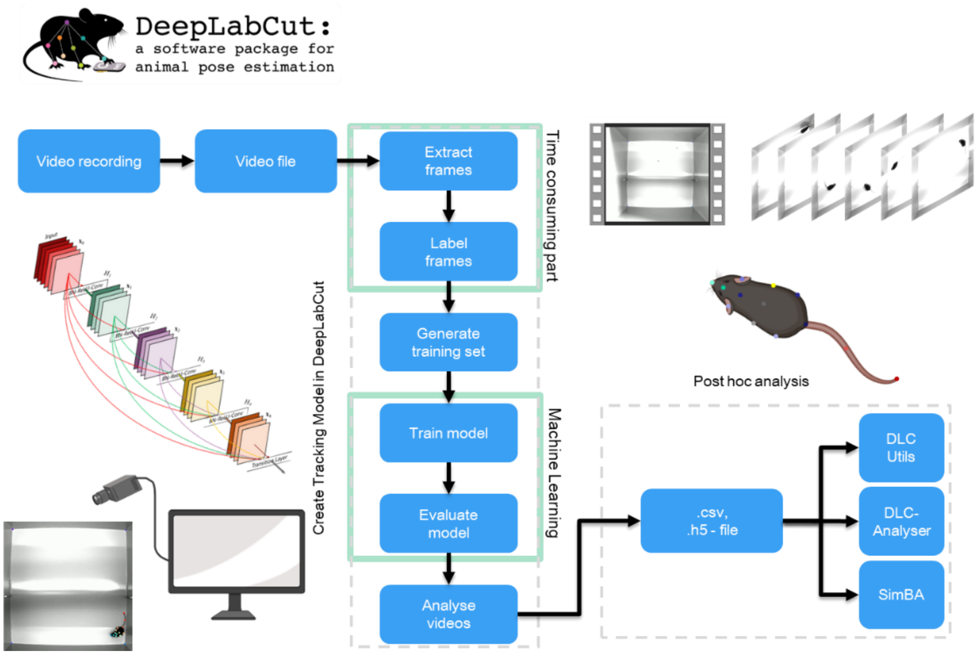

Fig. 1S: Schematic overview of the most important DLC-steps.

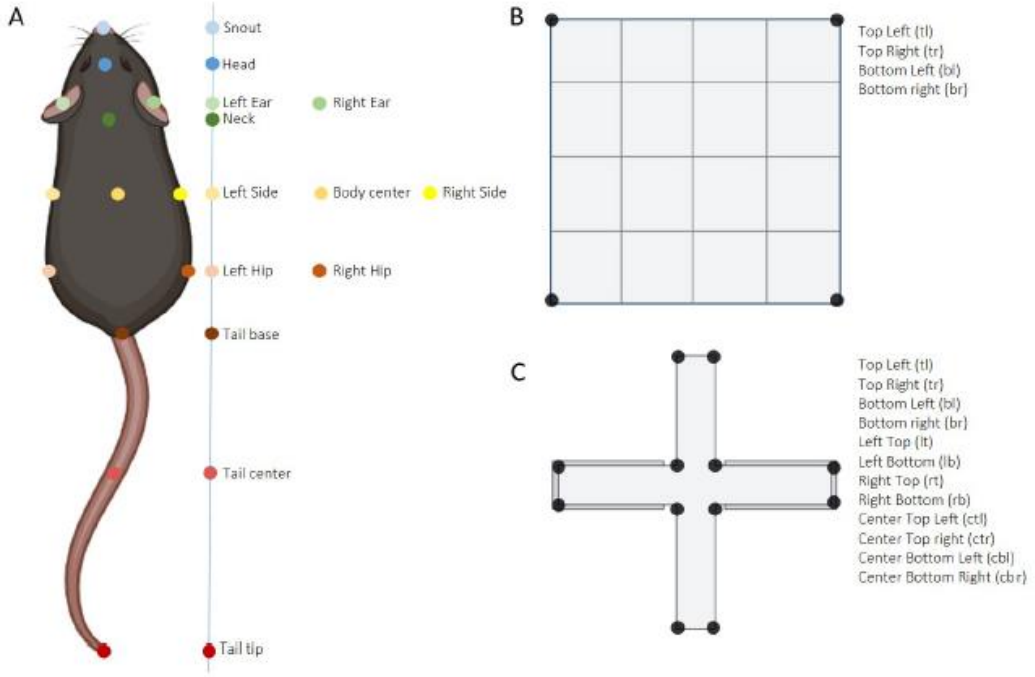

Fig. 2S: Overview of the labels used in the DLC network for pose estimation. To track the animal properly (A) 13 points of interest were labelled on the animal. Additionally, to predefine the regions of interest of the OF (B) and EPM (C), the arenas also had to be labelled. Abbreviations: DLC: DeepLabCut.

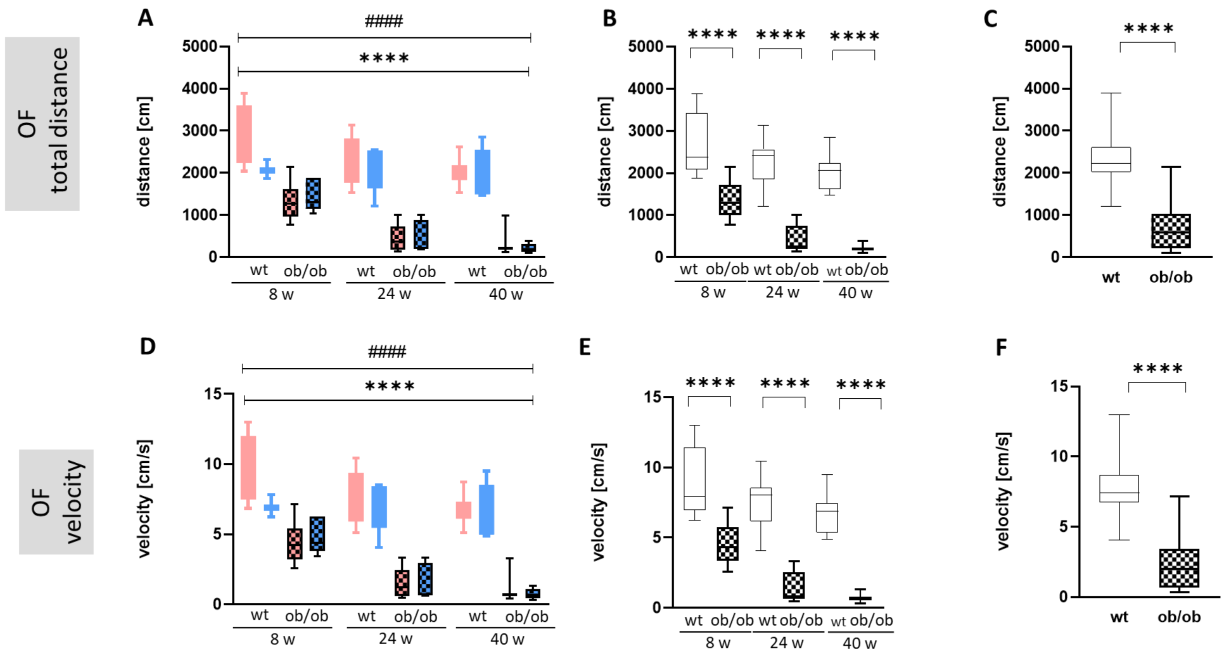

Fig. 3S: OF test analysis. Female (red) and male (blue) mice with different genetic backgrounds were analyzed at 8, 24, 40 weeks (w) of age. Analyses were done with the EthoVision software. Moved distance [cm] split up by (A) both sexes, genotypes (wild type (wt), ob/ob), and all ages, (B) both genotypes, all ages and (C) both genotypes. Mean velocity [cm/s] split up by (D) both sexes, genotypes, and all ages, (E) both genotypes, all ages and (F) both genotypes. Irrespective of sex, ob/ob mice show a significantly decreased locomotion compared to the wt littermates. The group size was as follows: n (wt) = 36, n (ob/ob) = 40 (A-C) and 39 (D-F). Significance of differences between the groups were tested by three- (A and D), two-way ANOVA with Sidak´s post hoc test for multiple comparisons (B and E) or unpaired Student-t-test (C and F). For A and D, analysis revealed a genotype and age effect (A: genotype $p<0.0001$, $F(1,64)=198.6$; age $p<0.0001$, $F(2,64)=17.57$; D: genotype $p<0.0001$, $F(1,64)=198.6$); age $p<0.0001$, $F(2,64)=17.66$. For A and D statistical significance was set at **** $p<0.0001$ for genotype effect and $^{####}$ $p<0.0001$ for age effect. For B and E, as well as C, and F. Statistical significance was set at **** $p<0.0001$.

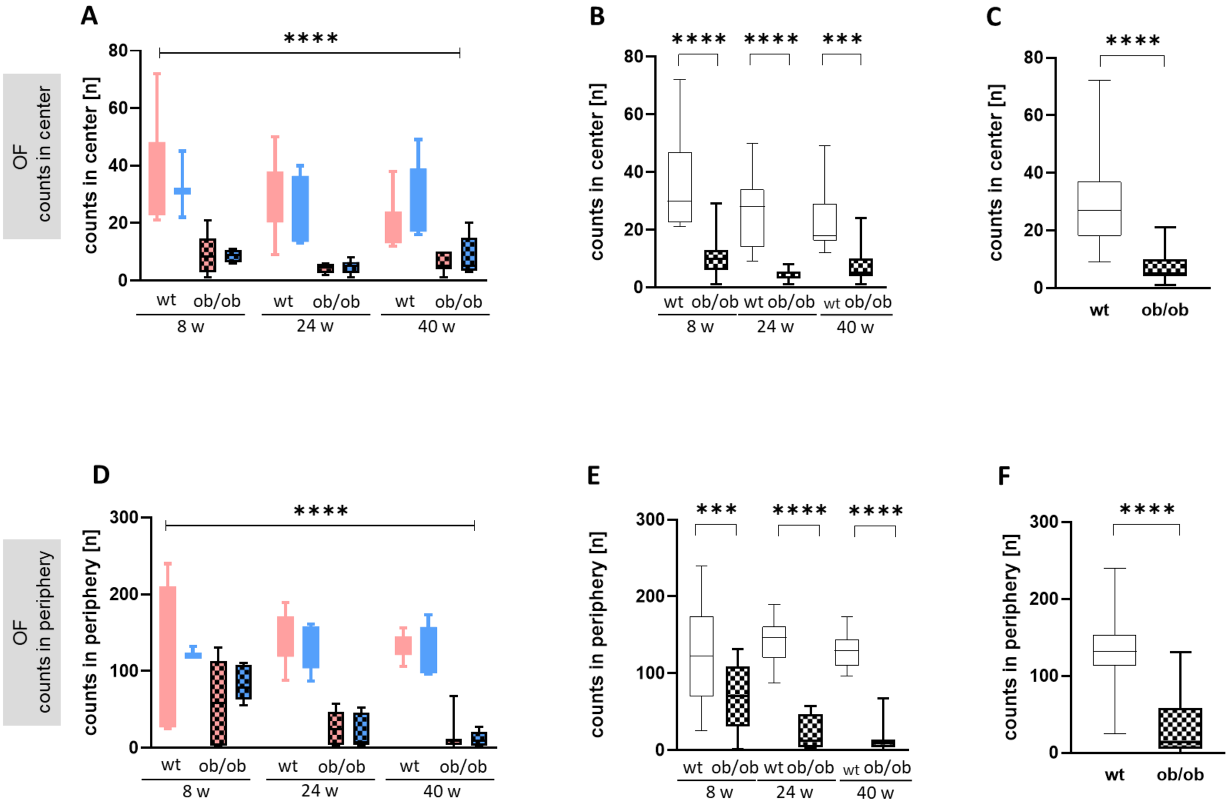

Fig. 4S: OF test analysis. Female (red) and male (blue) mice with different genetic backgrounds were analyzed at 8, 24, 40 weeks (w) of age. Analyses were done with the EthoVision software. Number of center counts [n] split up by (A) both sexes, genotypes (wild type (wt), ob/ob), and all ages, (B) both genotypes, all ages and (C) both genotypes. Number of periphery counts [n] split up by (D) both sexes, genotypes, and all ages, (E) both genotypes, all ages and (F) both genotypes. Irrespective of sex, ob/ob mice show a significantly decreased number of visits in the center compared to the wt mice. The group size was as follows: n (wt) = 36, n (ob/ob) = 39 (A-C) and 40 (D-F). Significance of differences between the groups were tested by three- (A and D), two-way ANOVA with Sidak's post hoc test for multiple comparisons (B and E) or unpaired Student-t-test (C and F). For A and D, analysis revealed a genotype effect (A: genotype $p<0.0001$, F(1,63=80.41; D: genotype $p<0.0001$, $F(1,64)=90.87$). For A and D statistical significance was set at **** $p<0.0001$ for genotype effect. For B and E as well as C and F Statistical significance was set at *** $p<0.001$, **** $p<0.0001$.

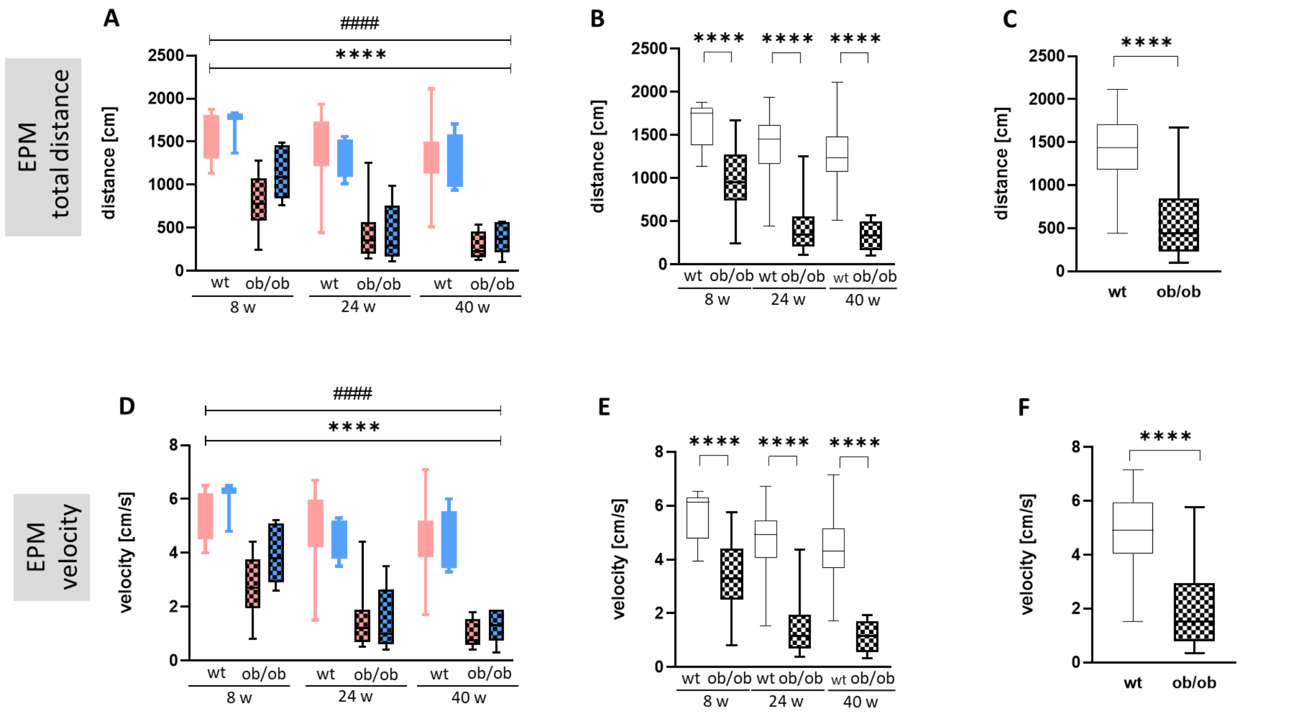

Fig. 5S: EPM analysis. Female (red) and male (blue) mice with different genetic backgrounds were analyzed at 8, 24, 40 weeks (w) of age. Analyses were done with the EthoVision software. Moved distance [cm] split up by (A) both sexes, genotypes (wild type (wt), ob/ob), and all ages, (B) both genotypes, all ages and (C) both genotypes. Mean velocity [cm/s] split up by (D) both sexes, genotypes, and all ages, (E) both genotypes, all ages and (F) both genotypes. Irrespective of sex, ob/ob mice show a significantly decreased locomotion compared to the wt littermates. The group size was as follows: n (wt) = 45, n (ob/ob) = 45 (except A and D, n = 44). Significance of differences between the groups were tested by three- (A and D), two-way ANOVA with Sidak's post hoc test for multiple comparisons (B and E) or unpaired Student-t-test (C and F). For A and D, analysis revealed a genotype and age effect (A: genotype $p<0.0001$, $F(1,77)=131.2$; age $p<0.0001$, $F(2,77)=15.49$; D: genotype $p<0.0001$, $F(1,77)=132.3$; age $p<0.0001$, $F(2,77)=16.03$. For A and D statistical significance was set at **** $p<0.001$ for genotype effect and $^{\#\#\#\#}$ $p<0.001$ for age effect. For B and E as well as C and F. Statistical significance was set at **** $p<0.0001$.

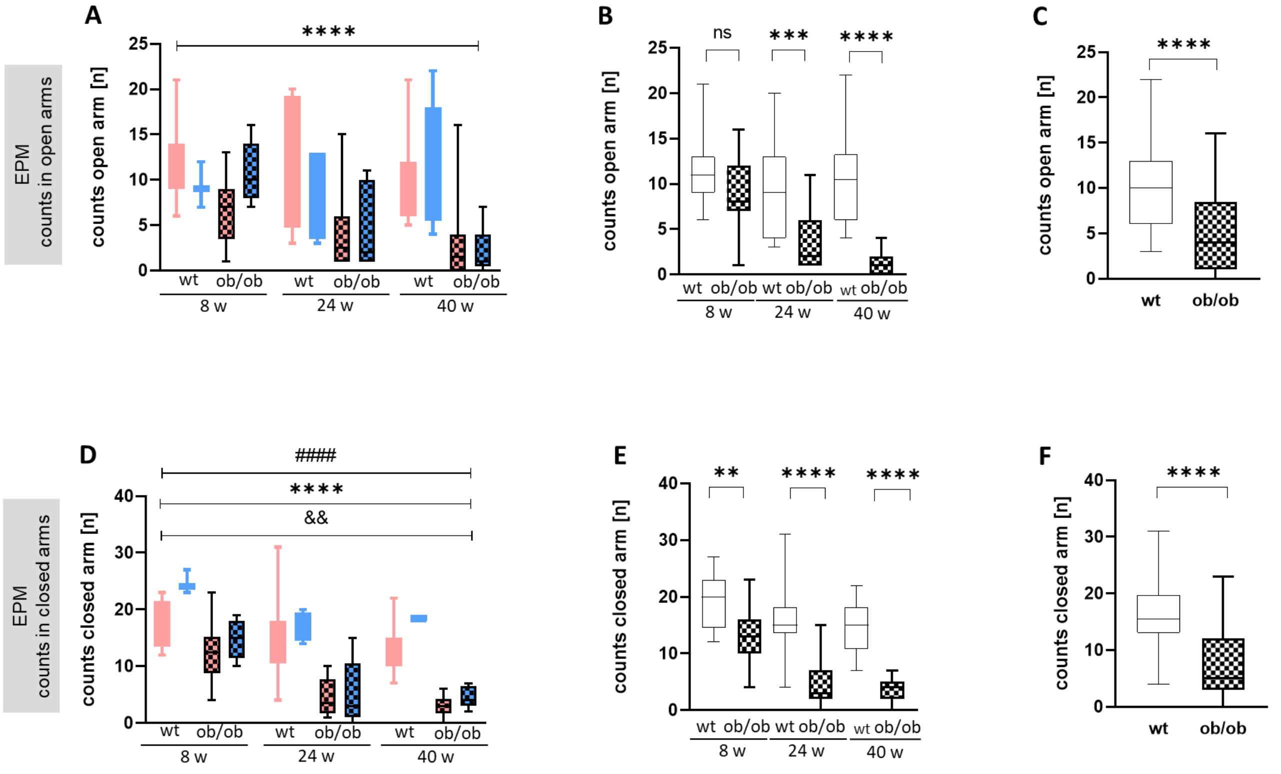

Fig. 6S: EPM analysis. Female (red) and male (blue) mice with different genetic backgrounds were analyzed at 8, 24, and 40 weeks (w) of age. Analyses were done with the EthoVision software. Number of counts [n] in the open arm split up by (A) both sexes, genotypes (wild type (wt), ob/ob), and all ages, (B) both genotypes, all ages and (C) both genotypes. Number of counts [n] in the closed arms split up by (D) both sexes, genotypes, and all ages, (E) both genotypes, all ages and (F) both genotypes. Irrespective of sex, ob/ob mice show a significantly increased aversion to the open arms compared to their corresponding littermates. The group size was as follows: n (wt) = 45 (A-C) or 44 (D-F), n (ob/ob) = 45 (A and C), 42 (B). Significance of differences between the groups were tested by three- (A and D), two-way ANOVA with Sidak's post hoc test for multiple comparisons (B and E) or unpaired Student-t-test (C and F). For A and D, analysis revealed a genotype effect (A: genotype $p<0.0001$, $F(1,78)=21.50$; D: genotype $p<0.0001$, $F(1,77)=105.5$), partly an age and gender effect (D: age $p<0.0001$, $F(2,77)=21.66$; gender $p=0.0024$, $F(1,77)=9.865$). For A and D statistical significance was set at **** $p<0.001$ for genotype effect, $^{####}$ $p<0.001$ for age and $^{\&\&}$ $p<0.01$ for gender effect. For B and E as well as C and F. Statistical significance was set at** $p<0.01$, *** $p<0.001$, **** $p<0.0001$.

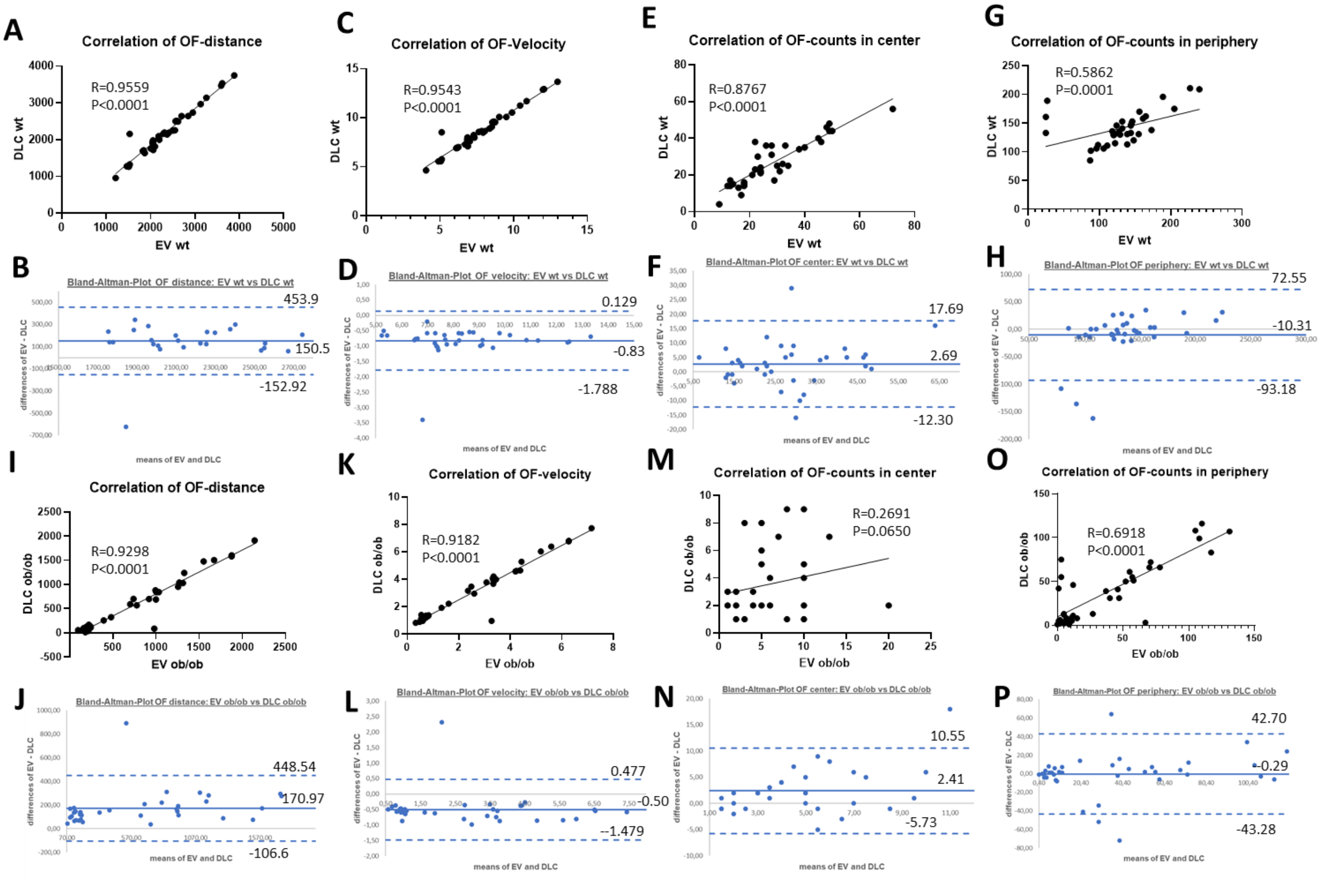

Fig. 7S: Spearman Correlation (A, C, E, G, I, K, M, O) and Bland-Altman-Plot (B, D, F, H, J, L, N, P) analysis of EV versus DLC for the **OF test** dataset of age- and sex-matched wt and ob/ob mice. (A, B and I, J) distance, (C, D and K, L) velocity, (E, F and M, N) counts in center, and (G, H and O, P) counts in periphery. For the Spearman Correlation the individual correlation coefficients and significances are given in the graph. For Bland-Altman Plot analysis the means ± 1.96*SD and the means of the differences of both softwares (EV and DLC) are given in the graph. Abbreviations: EV: EthoVision, DLC: DeepLabCut, ob/ob: leptin deficient mice, OF: Open Field, wt: wild type.

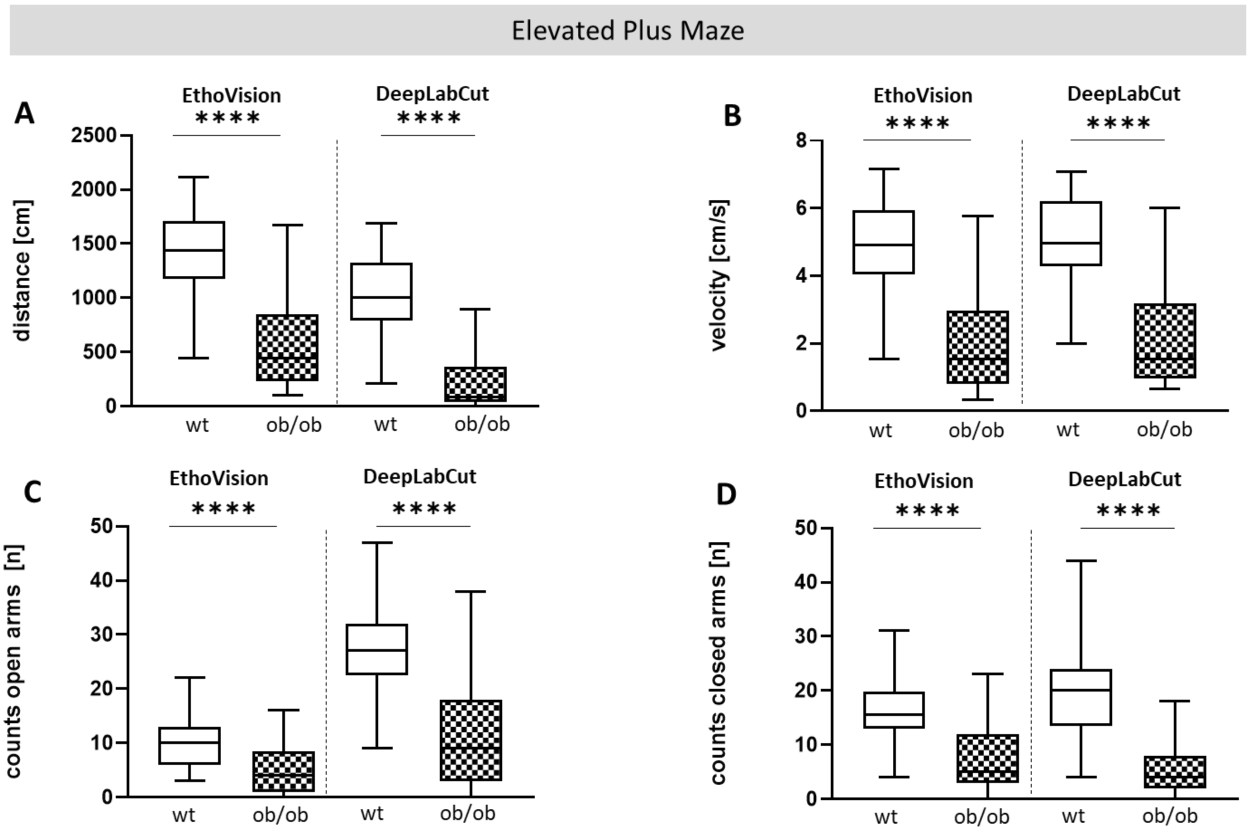

Fig. 8S: Comparative analysis with EV and DLC. EPM analysis of wt and ob/ob mice independent of sex and age. (A) Moved distance [cm], (B) mean velocity [cm/s], (C) number of visits (counts) in open arms [n], (D) number of visits (counts) in closed arms [n]. Significance of differences between the groups were tested by unpaired Student-t-test. Group size was as follows: n (wt) = 45 (EV) or 46 (DLC), n (ob/ob) = 45 (EV) or 39-44 (DLC). Statistical significance was set at **** $p<0.0001$; Abbreviations: ob/ob: leptin deficient mice, wt: wild type, EV: EthoVision, DLC: DeepLabCut.

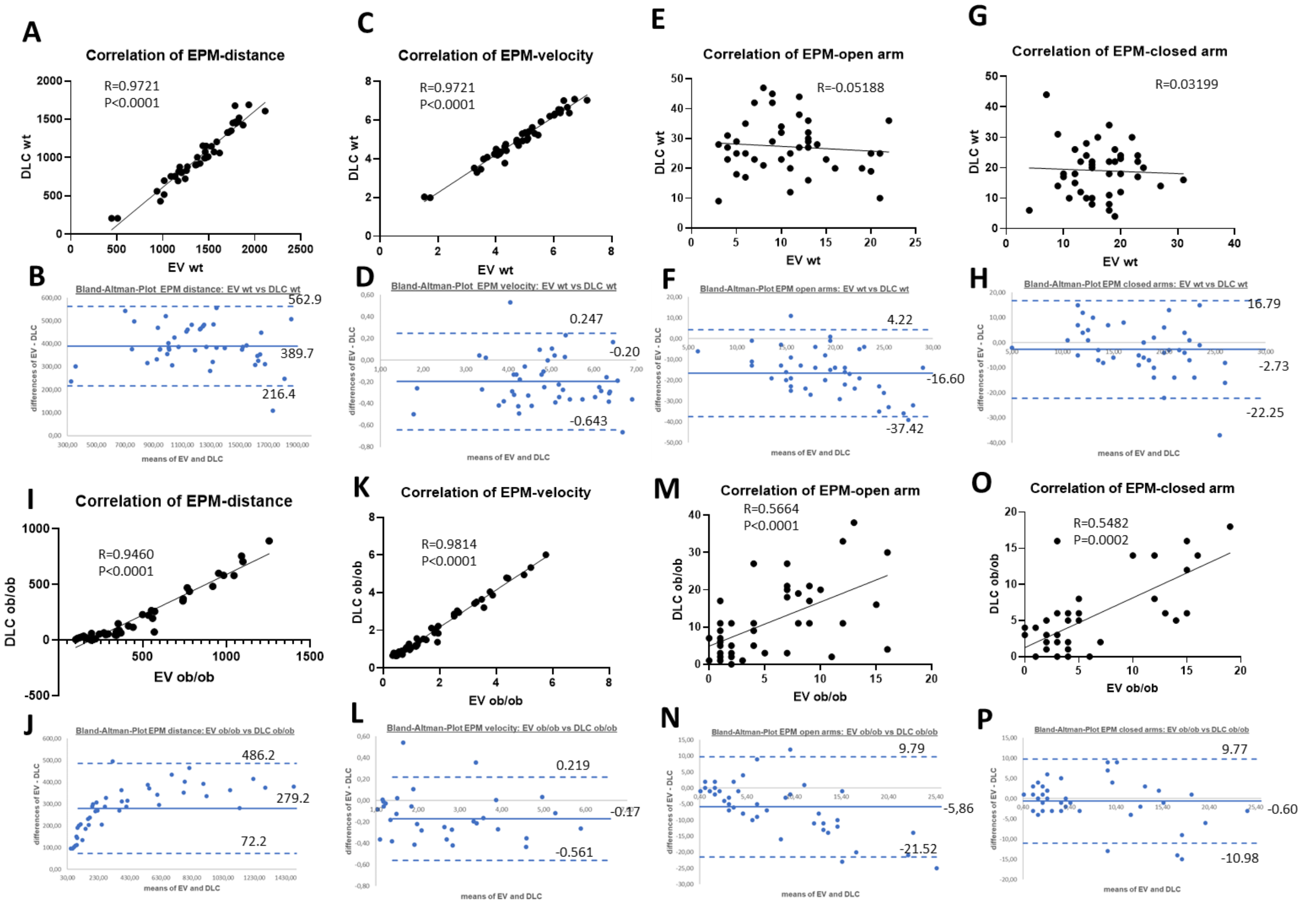

Fig. 9S: Spearman Correlation (A, C, E, G, I, K, M, O) and Bland-Altman-Plot (B, D, F, H, J, L, N, P) analysis of EV versus DLC for the **EPM** dataset of age- and sex-matched wt and ob/ob mice. (A, B and I, J) distance, (C, D and K, L) velocity, (E, F and M, N) counts in open arms, and (G, H and O, P) counts in closed arms. For the Spearman Correlation the individual correlation coefficients and significances are given in the graph. For Bland-Altman Plot analysis the means ± 1.96*SD and the means of the differences of both softwares (EV and DLC) are given in the graph. Abbreviations: EV: EthoVision, DLC: DeepLabCut, ob/ob: leptin deficient mice, EPM: Elevated Plus Maze, wt: wild type.
